# The effect of autoclave cycles on the mechanical properties and surface roughness of NiTi archwires: ex-vivo study

**DOI:** 10.1186/s12903-024-04877-4

**Published:** 2024-10-24

**Authors:** Amal Magdi El Shahawi, Amira Ahmed Aboalnaga

**Affiliations:** 1 Researcher at the Restorative and Dental Materials Department, Oral and Dental Research Institute at the National Research Centre, Cairo, Egypt; 2 Lecturer at the Department of Orthodontics and Dentofacial Orthopedics, Faculty of Dentistry, Cairo University, Cairo, Egypt

**Keywords:** Mechanical properties, Surface roughness, Clinical recycling, Rapid-cycle autoclaving, Universal-cycle autoclaving

## Abstract

**Objectives:**

To evaluate the effect of the universal and rapid autoclave cycles on the mechanical properties and surface roughness of nickel-titanium archwires following clinical use.

**Material and methods:**

Thirty-six NiTi archwires (0.016 × 0.022 inch) were equally divided into a control group (Group A) and 2 experimental groups (Group B & C). Wires in group A were tested in the “as-received” form. Wires in the two other groups were installed in patients mouth for 4 weeks, and then autoclaved using the rapid-cycle (Group B) or the universal-cycle (Group C). All wires were subjected to 3-point bending test to calculate the elastic limit, modulus of elasticity, spring-back, yield strength, resilience and toughness. Atomic force microscopy (AFM) was used for surface roughness qualitative and quantitative analysis.

**Results:**

Group B showed significantly higher values of elastic limit, modulus of elasticity, resilience, yield strength and toughness than the other two groups. No significant differences were detected between groups A and C (*P* > 0.05). Group B showed significantly lower average surface roughness than the other two groups, but no significant differences were detected between groups A and C (*P* > 0.05).

**Conclusions:**

The mechanical properties and surface roughness of clinically used NiTi wires were less affected by the universal-cycle than the rapid-cycle autoclaving. However, the difference between the effect of both autoclave cycles was diminutive.

**Clinical relevance:**

The mechanical properties and surface roughness of the tested NiTi wires were not notably altered by clinical use and autoclaving.

## Introduction

Since its introduction in the early 1970s, Nickel-titanium (NiTi) archwires have gained much popularity in clinical orthodontics worldwide as leveling and aligning wires. Owing to their sustained low force delivery and wide elastic working range, marked irregular teeth may be initially engaged without permanent archwire deformation nor the need for complex loops [1]. NiTi archwire alloys utilized in orthodontic practice have almost equiatomic compositions (55% Ni and 45% Ti) and are based on the NiTi intermetallic compound [2]. These alloys have two main phases; body-centered cubic Austenitic and hexagonal close-packed Martensitic with an intermediate R-phase. Reversible phase transformation occurs from one crystallographic phase to the other as in response to fluctuations of temperature and stress. Austenite is a low stress/high temperature form whereas Martensite is a high stress/low temperature form [3].

In 1997, Kusy [4] introduced a classification for NiTi wires based on the dominant crystallographic phase; Austenite-active (equivalent to superelastic), Martensite-active (equivalent to shape memory), and Martensite-stabilized (equivalent to non-superelastic Nitinol, where the wire has a heavily work-hardened micro-structure). When force is applied to Austenitic-active wires upon ligating displaced teeth, small islands of martensitic crystalline structure are established within the predominantly austenitic wire allowing secure fit into the bracket slot. As the teeth align, the martensitic areas become restored by austenite. Superelasticity of the NiTi archwire refers to the process of applying low and constant force with a plateau during its loading or unloading (Austenitic-Martensitic transformation). As regards Martensitic-active wires, they exhibit a thermally induced shape memory effect in which they undergo structural changes when heated above its temperature transition range (TTR). At room temperature, the predominantly martensitic alloy is flexible and easily engaged to severely displaced teeth. Upon installation of the archwire intra-orally, the oral cavity raises the temperature of the deformed martensitic archwire increasing the ratio of the austenite phase, thereby recovering the original archform and becoming stress-resistant. The remarkable mechanical properties of NiTi wires and their comparatively high cost have compelled many clinicians to recycle these wires formerly [5, 6].

The current delivery of dental care is not sustainable due to rising costs, increasing demands, and the high environmental burden. Single use materials and instruments contribute significantly to the environmental carbon footprint of dentistry [7]. Archwire recycling may be one method to reduce the orthodontic practice overhead and enhance waste management, endorsing sustainability and promoting green dentistry. Besides, clinicians often require the use of the same NiTi archwires during a single patient treatment in many situations, as in case of re-leveling and alignment or in the finishing stage. Not to mention that manufacturers' instructions on the wire packages often emphasize the necessity of sterilization before clinical application if additional protection is required [8].

Many studies [9–15] evaluated the effect of recycling on the mechanical properties of NiTi archwires, however the results were controversial. Kapila et al. [11] assumed that Nitinol and superelastic NiTi wires manifested reduced elasticity and increased stiffness following clinical use and heat sterilization. Nevertheless, the detected changes in load-deflection characteristics of the wires were relatively trivial and hence its clinical significance were contentious. Meanwhile, later studies conducted by Smith et al. [10], Crotty et al. [12] and Pernier et al. [8] argued that autoclave sterilization did not affect the mechanical properties of superelastic NiTi wires.

As regards to surface topography, scarce evidence is found regarding the effect of recycling on surface roughness [13, 14]. Previous research [13, 14] pointed out that NiTi wires revealed greater surface roughness in the form of indentations and pitting subsequent to recycling.

There appear to be limited clinical studies that essentially revealed the effect of clinical recycling (intra-oral installation of the archwires followed by autoclaving) on the physical and mechanical properties of NiTi wires. The majority of studies either tested the effect of sterilization on new archwires which is an impractical approach that is not commonly performed in clinics, or in-vitro studies that are unable to simulate the oral environment in which the orthodontic archwires are supposed to survive and function [8]. Accordingly, the aim of our study was to evaluate the effect of the universal- and rapid-cycle autoclaving on the mechanical properties and surface roughness of nickel-titanium archwires following clinical use, in order to evaluate the efficacy of its recycling. Our null hypothesis was that both the universal- and rapid-cycle autoclaving have no effect on the mechanical properties and surface roughness of the tested nickel-titanium archwires.

## Material and methods

Thirty-six NiTi archwires (American Orthodontics, Wisconsin, USA) with a cross-Sect. 0.016 × 0.022 inch from a single batch were investigated in this study. Wires were randomly distributed into a control group (Group A) and 2 experimental groups (Group B & C). Wires in group A were tested in the “as-received” form. Wires in the two experimental groups were installed intra-orally for 4 weeks, and then retrieved, examined for any permanent deformation, rinsed under clean running water and dried. Wires in group B were placed unwrapped (as recommended by the autoclave manufacturer) on an instrument rack and autoclaved through a single rapid sterilization cycle (4 min, 134°C, 273.2°F). Wires in group C were packaged in sterilization bags, one archwire per bag and autoclaved through a single universal sterilization cycle (20 min, 121°C, 249.8°F). The autoclave used in this study was (Anthos A-17 Autoclave, Italy).

### Subjects

Twenty-four subjects who met the following inclusion criteria were selected for this study: Male or female patients ranging from 16 to 30 years old, healthy periodontal condition and good oral hygiene, no history of para-functional habits, and exhibiting mild to moderate crowding. All patients were treated with pre-adjusted 22-inch bracket slot (American Orthodontics, Inc., Florida, USA). Patients were in the leveling and alignment stage of a non-extraction orthodontic treatment, in which the operator (second author) routinely used the tested 0.016 × 0.022 NiTi archwires. Patients were given the regular orthodontic treatment instructions, which were to maintain optimum oral hygiene and thorough toothbrushing, avoid hard and sticky foods, and maintain their follow up visit after 4 weeks.

Based on a previous study [12], a sample size of 11 archwires per group was sufficient to detect a large effect size (f) = 0.88, with an actual power (1-β error) of 0.8 (80%) and a significance level (α error) 0.05 (5%) for two-sided hypothesis test.

### Test methods

Each archwire was cut into 3 segments; 2 straight distal segment and one anterior curved segment. The distal segments were used to derive the mechanical properties and the anterior segment was used to evaluate the surface roughness.

### Mechanical properties testing

**Fig. 1:**
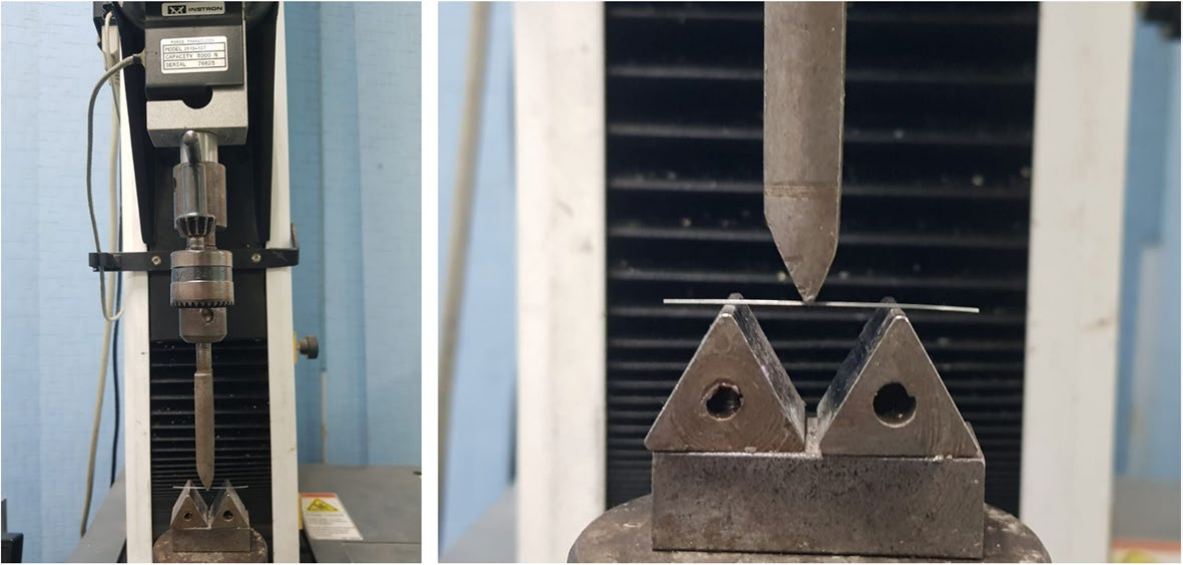
Archwire segments subjected to 3-point bending test using a computer controlled universal testing machine

All wires were subjected to 3-point bending test as described by Miura et al. [16] on a computer controlled universal testing machine (Model 3345; Instron Industrial Products, Norwood, MA, USA) with a loadcell of 5kN. Data were recorded using computer software (Instron® Bluehill Lite Software). The setup included a specially constructed fixture comprising two poles placed 12 mm apart on a stage attached to the lower jaw of the machine. Compressive force was applied at a crosshead speed of 0.5 mm/min by means of a steel rod of 0.6 mm radius with bi-bevelled chisel end placed midway between the two poles (Fig. 1), and the load required to produce 3.1 mm deflection was recorded [16]. The stress-strain curves were recorded using the computer software (Instron® Bluehill Lite Software).

The elastic limit (EL), modulus of elasticity (E), spring-back, yield strength (YS), resilience (R), and toughness (T) were calculated for each wire group. Elastic limit is defined as the maximum stress a material can withstand without permanent deformation. Young’s modulus of elasticity (E) delineates the stiffness of a material, which is measured by the slope of the linear elastic portion of the stress/strain curve. The modulus of elasticity (E) was calculated using the following equation:

$${\text{E = L}}^3\text{m}/\;4\text{bd}^3(\text{GPa})$$

where L is the wire span (mm), b is the width of specimen (mm), d is the depth of specimen (mm), and m is the slope of the straight-line portion of the stress strain curve (N/mm of deflection).

Spring-back is the ratio of proportional limit to modulus (PL/E) after unloading and it represents the working range of the archwire. Yield strength (YS) is defined as the highest stress experienced within the material just before it yields in a three-point flexural test. It is was calculated with the following equation:

$$\text{YS }=3\text{FL}/\;2\text{bd }^2$$

where; F is the maximum load at the point of fracture, L is wire span, b is the width of the sample (mm) and d is the depth of specimen (mm).

Resilience is the area under the stress/strain curve within the elastic portion and represents the energy needed for tooth movement when the wire is loaded to its elastic limit. Resilience (R) was calculated according to the following formulae:

$$\text{R }=\text{ YS}^2/\;2\text{E}$$

where; YS is the yield strength and E is the modulus of elasticity.

Toughness is the ability of a material to absorb elastic energy and to deform plastically before fracturing, measured as the total area under a load deflection curve. Calculation of all former values was guided by the current American National Standard/American Dental Association Specification No. 32 for Orthodontic Wires (2000) [17, 18].

### Surface roughness testing

Atomic force microscopy (AFM) was used for surface roughness qualitative and quantitative analysis [19, 20]. Wire segments 5 mm in length were evaluated using the AFM (Anton Paar GmbH-Tosca200 AFM, Germany) employing the tapping mode 500 µm increment with a rate of 1 line per second [19]. Software analysis performed by (Mountains 8.2 Software-Digital Surf, Besancon, France) was employed to calculate the following surface roughness variables: Average surface roughness (Sa), Root mean square height (Sq) which is equivalent to the standard deviation of the heights, Skewness value (Ssk) which represents the degree of bias of the roughness shape, Kurtosis value (Sku) which is a measure of the sharpness of the roughness profile, Maximum peak height (Sp) which is the height of the highest peak within the defined area, Maximum pit depth (Sv) which is the depth of the deepest pit within the defined area and Maximum height (Sz) which is the sum of the largest peak height value and the largest pit depth value within the defined area (Sp + Sv).

## Statistical analyses

A standard software package (SPSS version 20, Chicago, Ill) was used for data analysis. Shapiro-Wilk test was used to test the normality hypothesis of variables. Variables of mechanical properties and surface roughness were described by the mean, standard deviation (SD), standard error (SE), 95% confidence interval of the mean values and range (Minimum – Maximum). For normally distributed variables, ANOVA test followed by Bonferroni test was used for intergroup comparisons. In case of non-normally distributed variables, Kruskal-Wallis test followed by Dunn test were used for intergroup comparisons. In all the above statistical tests, a probability value of 0.05 was considered significant.

## Results

### Mechanical properties

**Fig. 2:**
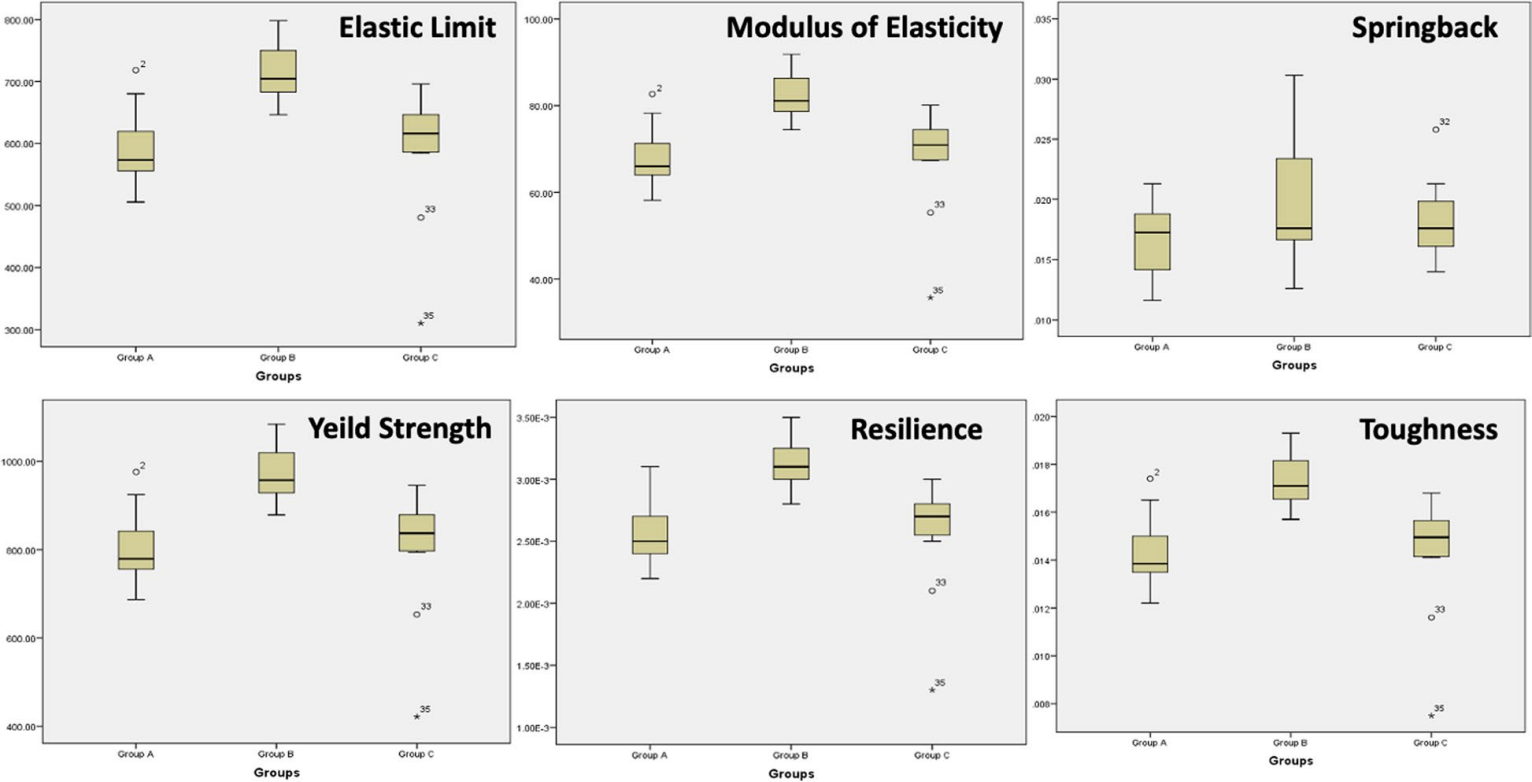
Box and whisker plot comparing the mechanical properties between the three groups

Results of the elastic limit (EL), modulus of elasticity (E), spring-back, yield strength (YS), resilience (R), and toughness (T) of the three groups are shown in Table 1 and Fig. 2. Results of all variables showed a parametric distribution. Elastic limit, modulus of elasticity, resilience, yield strength, toughness showed significant differences between the groups. Group B showed significantly higher values than the other two groups. There were significant differences between groups A and B, and groups B and C (*P* < 0.001), but no significant difference were detected between groups A and C (*P* > 0.05). Regarding spring back, no significant difference was detected between the three groups (*P* > 0.05).

**Table 1:** Means, standard deviations (SD), standard error (SE), 95% confidence interval of the mean values, range (Minimum – Maximum) and P values of the mechanical properties of the three wire groups (ANOVA and Bonferroni post hoc tests)

**Mechanical properties**		**Mean**	**SD**	**SE**	**95% Confidence interval for mean**		**Range**		***P* Value**
					**Lower bound**	**Upper bound**	**Minimum**	**Maximum**	
**Elastic limit (MPa)**	**Group A**	593.02^a^	61.08	17.63	554.21	631.83	505.51	718.21	0.00018***
	**Group B**	717.37^b^	50.28	14.52	685.42	749.31	646.47	797.96	
	**Group C**	589.39^a^	102.92	29.71	524.00	654.78	310.21	695.81	
**Modulus of Elasticity (GPa)**	**Group A**	68.27^a^	7.03	2.03	63.80	72.74	58.19	82.68	0.00018***
	**Group B**	82.58^b^	5.79	1.67	78.91	86.26	74.42	91.86	
	**Group C**	67.85^a^	11.85	3.42	60.32	75.38	35.71	80.10	
**Spring back**	**Group A**	0.0166	0.0030	0.0009	0.0147	0.0186	0.0116	0.0213	0.18882
	**Group B**	0.0196	0.0050	0.0014	0.0164	0.0228	0.0126	0.0303	
	**Group C**	0.0181	0.0033	0.0010	0.0160	0.0202	0.0140	0.0258	
**Yield strength (MPa)**	**Group A**	805.81^a^	83.00	23.96	753.07	858.55	686.91	121.90	0.00018***
	**Group B**	974.78^b^	68.33	19.72	931.37	1018.19	878.44	99.30	
	**Group C**	800.89^a^	139.85	40.37	712.03	889.74	421.53	122.40	
**Resilience (J)**	**Group A**	0.002583^a^	0.000269	0.000078	0.002412	0.002754	0.002200	0.003100	0.00021***
	**Group B**	0.003125^b^	0.000230	0.000066	0.002979	0.003271	0.002800	0.003500	
	**Group C**	0.002558^a^	0.000456	0.000132	0.002268	0.002848	0.001300	0.003000	
**Toughness (J)**	**Group A**	0.0144^a^	0.0015	0.0004	0.0134	0.0153	0.0122	0.0174	0.00017***
	**Group B**	0.0174^b^	0.0012	0.0003	0.0166	0.0181	0.0157	0.0193	
	**Group C**	0.0143^a^	0.0025	0.0007	0.0127	0.0158	0.0075	0.0168	

Group A: Control “as-received” wires, Group B: Short cycle autoclaved wires, Group C: Universal cycle autoclaved wires

^a,b^Different superscript letters indicate a statistically significant difference (*P* < 0.05), *** *P* < 0.001

### Surface roughness

**Fig. 3:**
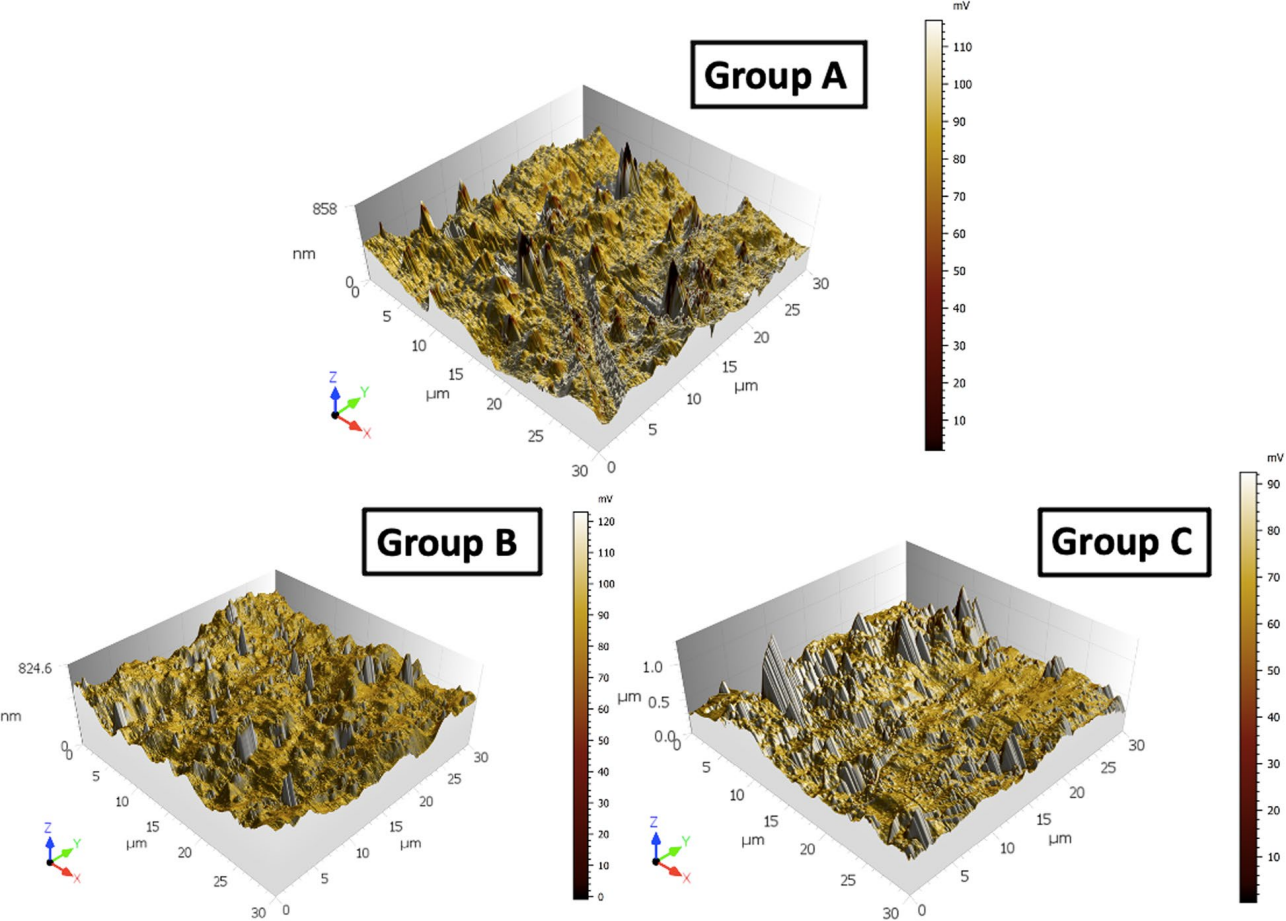
Tapping-mode AFM topographical 3D images of the wire groups demonstrated in nanometer (nm). Group A: Control “as-received” wires, Group B: Rapid-cycle autoclaved wires, Group C: Universal-cycle autoclaved wires

**Fig. 4:**
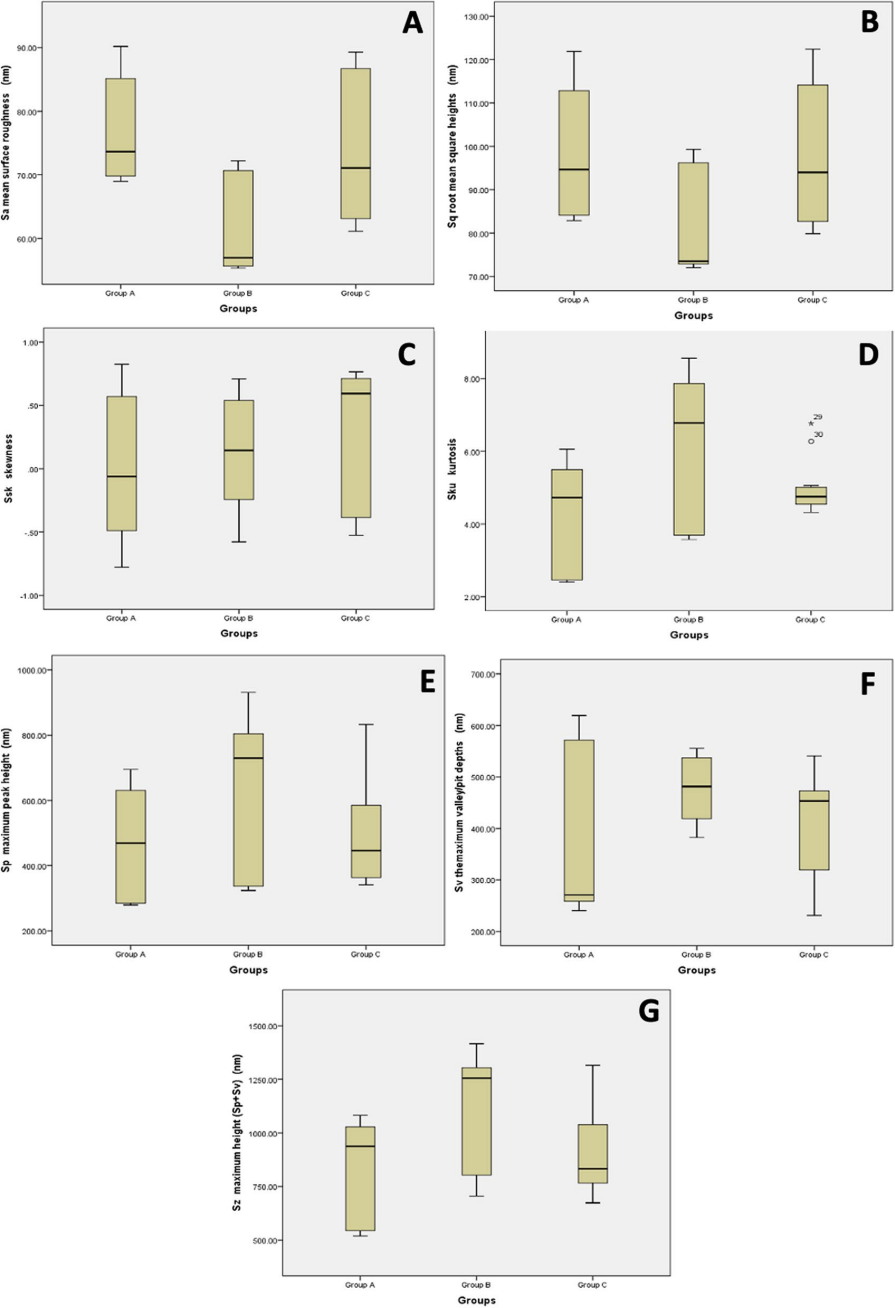
Box and whisker plot comparing the surface roughness variables between the three groups. **A** Average surface roughness (Sa), **B** Root mean square height (Sq), **C** Skewness value (Ssk), **D** Kurtosis value (Sku), **E** Maximum peak height (Sp), **F** Maximum pit depth (Sv) and **G** Maximum height (Sz)

Results of surface roughness analysis of the three groups are shown in Table 2 & Figs. 3 & 4. Sa, Sz, Sku and Sp showed a non-parametric distribution, while Sq, Ssk and Sv showed a parametric distribution. Average surface roughness (Sa) and Root mean square height (Sq) showed significant differences between the groups. Group B showed significantly lower values than the other two groups. There were significant differences between groups A and B, and groups B and C (*P* < 0.001), but no significant differences were detected between groups A and C (*P* > 0.05). Regarding Skewness (Ssk), Kurtosis (Sku), Maximum peak height (Sp), Maximum pit depth (Sv) and Maximum height (Sz), no significant differences were detected between the groups (*P* > 0.05).

**Table 2:** Means, standard deviations (SD), standard error (SE), 95% confidence interval of the mean values, range (Minimum – Maximum) and P values of the surface roughness of the three wire groups (ANOVA and Post Hoc tests, Kruskal-Wallis and Dunn tests)

**Surface roughness variables**		**Mean**	**SD**	**SE**	**95% Confidence interval for mean**		**Range**		***P* Value**
					**Lower bound**	**Upper bound**	**Minimum**	**Maximum**	
**Average surface roughness (Sa) (nm)**	**Group A**	76.76^a^	7.96	2.30	71.70	81.82	68.98	90.18	0.00218***
	**Group B**	61.31^b^	7.50	2.17	56.54	66.08	55.32	72.21	
	**Group C**	73.71^a^	11.07	3.19	66.68	80.75	61.14	89.31	
**Root mean square height (Sq) (nm)**	**Group A**	98.25^a^	14.55	4.20	89.00	107.50	82.87	121.90	0.00875***
	**Group B**	81.18^b^	12.20	3.52	73.42	88.93	72.03	99.30	
	**Group C**	97.81^a^	16.15	4.66	87.55	108.07	79.85	122.40	
**Skewness (Ssk)**	**Group A**	0.02	0.57	0.16	-0.34	0.38	-0.78	0.83	0.40282
	**Group B**	0.11	0.43	0.12	-0.16	0.39	-0.58	0.71	
	**Group C**	0.30	0.55	0.16	-0.05	0.65	-0.53	0.77	
**Kurtosis value (Sku)**	**Group A**	0.02	0.57	0.16	-0.34	0.38	-0.78	0.83	0.08246
	**Group B**	0.11	0.43	0.12	-0.16	0.39	-0.58	0.71	
	**Group C**	0.30	0.55	0.16	-0.05	0.65	-0.53	0.77	
**Maximum peak height (Sp) (nm)**	**Group A**	468.28	161.48	46.62	365.67	570.88	279.00	695.10	0.16340
	**Group B**	639.76	238.59	68.88	488.16	791.35	323.40	931.40	
	**Group C**	500.12	169.33	48.88	392.53	607.70	340.90	833.20	
**Maximum pit depth (Sv) (nm)**	**Group A**	370.93	162.44	46.89	267.73	474.14	240.20	619.40	0.10657
	**Group B**	473.06	61.64	17.79	433.90	512.22	382.20	555.40	
	**Group C**	407.67	99.93	28.85	344.17	471.16	231.00	541.20	
**Maximum height (Sz) (nm)**	**Group A**	839.23	232.96	67.25	691.21	987.24	519.20	1082.00	0.07133
	**Group B**	1112.81	268.52	77.52	942.20	1283.42	705.60	1416.00	
	**Group C**	907.67	212.09	61.23	772.91	1042.42	673.70	1315.00	

Group A: Control “as-received” wires, Group B: Short cycle autoclaved wires, Group C: Universal cycle autoclaved wires

^a,^^b^Different superscript letters indicate a statistically significant difference (*P* < 0.05), *** *P* < 0.001

## Discussion

As a consequence of both cost and retention of the elastic properties subsequent to clinical usage, clinicians contemplated recycling of NiTi wires. The present ex-vivo study is distinguishable from previous research in that it compared and evaluated the properties of NiTi wires following clinical use and sterilization by two different autoclave cycles. Tested wires were austenitic superelastic thermostable NiTi wires, providing moderate force levels according to the claim of the manufacturer (NT3-SE®, American Orthodontics, Sheboygan, WI, USA), with an elemental composition of (50% Ni—48% Ti -2% Cr) [21].

The three-point bending test implemented in the current study is considered a standard method for appraising archwires’ flexural properties, providing insights on its clinical behavior [22, 23]. Tested wire samples are subjected to complex flexural stresses imitating clinical situations; in which tensile stresses develops on one side of the wire, compressive stresses develop on the other, and in between lies the neutral axis with nill stresses.

Upon explicating the results, significant differences were found in the mechanical properties among the wire groups. Rapid-cycle autoclaved wires showed significantly greater elastic limit and resilience than both as-received and universal-cycle autoclaved wires. Meanwhile, it revealed greater modulus of elasticity indicating greater wire stiffness. The mechanical properties of the universal-cycle autoclaved wires were intimately comparable to the as-received wires.

The negligible influence of universal-cycle autoclaving can be ascribed to several factors. Initially, it can be attributed to the thermostability and crystallographic microstructure of the NiTi wires studied. A former study [8] compared the combined effect of two months immersion in artificial saliva followed by universal-cycle autoclaving on 0.016-inch superelastic NiTi versus Copper NiTi wires. They recognized some loss of superelasticity in both wire groups as perceived from their load-deflection graphs. However, the effect of autoclaving was more pronounced in Copper NiTi wires owing to its thermosensitive nature as explained by the authors. Another study conducted by Lee and Chang [24] studied the effect of wire immersion in artificial saliva followed by universal-cycle autoclaving on Martensitic versus Austenitic NiTi archwires. They inferred that the three bending properties of recycled austenitic NiTi wires did not significantly alter, while the martensitic NiTi wires demonstrated a significant loss of pseudoelasticity. The authors explained the results due to the lower transition temperature range of the austenite type which is less affected by the higher temperatures. Another factor that could influence the effect of the sterilization method is the temperature of the procedure; rapid-cycle autoclaving (134°C) employs higher temperature than the universal-cycle (121°C). Kapila et al. [11] concluded that clinically recycled 0.016-inch NiTi wires followed by dry heat sterilization (235°C) have demonstrated significantly reduced pseudoelasticity and increased stiffness. Upon comparing the effect of dry heat (160°C) versus steam sterilization (121°C) on three different brands of as-received 0.016-inch superelastic NiTi wires, Alavi et al. [25] deduced that both sterilization methods reduced the superelastic ratio and exerted forces of the three tested wires, yet with a slightly greater depletion following dry-heat sterilization. Similarly, Alavi and Sinaee [26] conducted a parallel study on β-titanium alloy wires. They denoted that dry heat sterilization increased the stiffness of the tested wires, in contrast to autoclaving which did not alter the load-deflection characteristics of similar wires. These findings may be assigned to the higher temperature and longer process time of the dry heat versus steam sterilization. Despite the previous findings, such minor changes in the load-deflection characteristics had humble clinical impact [9, 11, 25, 26]. Mayhew and Kusy [6] proposed that changes in the tensile or flexural properties of archwires less than or equal to 10% would be clinically insignificant.

Several previous studies defended that the diverse sterilization regimes didn’t impact the mechanical properties of NiTi wires [6, 8, 12, 13]. However, these studies limited their inspection to the effects of sterilization procedures on new wires or wires which were immersed in artificial saliva. It must be appreciated that archwires which were recycled following installation in the oral cavity, being exposed to saliva and cyclic stresses from functional demands, would essentially display different mechanical characteristics. It’s worth mentioning that Kapila et al. [11] argued that the majority of the mechanical changes detected were ascribable to clinical recycling rather than the sterilization method. Another element which might have influenced the recycling effect on the mechanical properties is the cross-section of the archwires. Studies absolutely depreciating the effect of sterilization examined rectangular-cross section NiTi archwires [6, 8, 12, 13], on the other hand, all studies which reported a negative influence of recycling used 0.016-inch NiTi wires which are inherently less rigid [9, 11, 25].

One of the most chief problems that would probably encounter recycled NiTi wires is breakage due to repeated cyclic loading. Therefore, measurement of yield strength and toughness would give a good indication about the amount of elastic energy to be absorbed by the wires before they deform plastically and fracture. Rapid-cycle autoclaved wires have shown significantly increased yield strength and toughness than the universal-cycle autoclaved wires which were comparable to the as-received wires. A previous study conducted by Staggers and Margeson [27] examined the effect of a single autoclave cycle (121°C, 20 min) versus five cycles on new superelastic NiTi wire segments. They revealed that autoclaving produced a significant increase in the tensile strength of the archwires, with no significant difference between one and five cycles. Likewise, Lee et al. [13] reported a non-significant elevated fracture resistance of NiTi wires after clinical recycling and long-cycle autoclaving. One can postulate that autoclave sterilization might introduce a positive input on the strength of NiTi archwires if any.

Based on our findings, it can be inferred that the mechanical properties of the tested NiTi wires were relatively less affected by the universal-cycle compared to short-cycle autoclaving, most probably owing to the higher temperature of the latter. Miura et al. [16] inferred that austenite NiTi wires display clear changes in their crystal structure when sterilized at temperatures above 60 °C, which might be responsible for the minor changes in their mechanical properties reaching to complete loss of its superelasticity when sterilized for 10 min at 600 °C.

However, the difference between both autoclave cycles detected in the current study was diminutive. Moreover, the springback which is a clinical measure of the force required to cause biological tissue response, was statistically insignificant between the groups augmenting the fact that clinical recycling and post-sterilization changes cannot not be judged as deleterious to wire function. Our study certifies the results achieved earlier by Khier et al. [28], which surmised that heat treatment up to 500°C for 10 min caused negligible significant changes in the bending behavior of superelastic NiTi alloys.

The oral environment in which the orthodontic wires function represents a corrosive domain, introducing a variety of masticatory forces, oral hygiene status, pH and mouth temperature fluctuations in addition to the oral flora [29]. Scarce evidence about the effect of clinical recycling on the surface topography of NiTi wires exist. The current study utilized the atomic force microscopy (AFM) that provides a three-dimensional quantitative and qualitative assessment of the archwires’ micromorphology [18].

Upon interpreting our results, it seems that surface roughness was not notably altered by clinical use and autoclaving. Surprisingly, there was a significant decrease of about (-15 nm) in the average surface roughness (Sa) and root mean square height (Sq) of NiTi wires autoclaved using the rapid-cycle when compared to the universal-cycle. However, the detected change is extremely minimal thereby clinically ineffective. As regards all other qualitative and quantitative variables of surface roughness, they were unchanged following both autoclave cycles. Based on the classification of Bourauel et al. [30], archwires showing average surface roughness (Sa) values less than 200 nm possess a smooth surface finish. Thereby, it can be concluded that clinically used NiTi archwires retained their smooth surface finish following autoclaving using the rapid-cycle (Sa = 61.31 nm) and universal-cycle (Sa = 73.71 nm). Correspondingly, Pernier et al. [8] tested the effect of sterilization on the surface topography of new wires using AFM, and pointed out a negligible increase in the Sa of about 50 nm. Another research conducted by Ghazal et al. [29] studied the effect of oral environment on the surface topography of retrieved superelastic NiTi wires following clinical use for 30 days. Their results showed an increase of 25.74 nm in Sa based on AFM analysis. Both former studies deduced that tested archwires retained an acceptable surface topography. Nevertheless, one should bear in mind that alteration of archwires’ surface topography secondary to clinical recycling seems to differ from one brand to another according to the manufacturers’ production techniques [13, 15, 30, 31].

## Limitations of the study

The study was restricted to one brand of superelastic NiTi wires, therefore the conclusions cannot be generalized on all commercially available NiTi archwires. Further studies should be conducted on various archwire products to serve as a clinical guide for orthodontists. Secondly, the study did not differentiate between the results due to clinical use and sterilization method, each step solely. The outcomes clarified in the study was due to the combined effect of clinical use and sterilization since the authors believed that clinicians will not need to use NiTi wires following a single former step (clinical use/sterilization) in most circumstances.

## Conclusions

1. The mechanical properties and surface roughness of the clinically used superelastic NiTi wires were less affected by the universal-cycle versus short-cycle autoclaving. However, the difference between both autoclave cycles is diminutive.
2. Elastic limit, modulus of elasticity, resilience, yield strength and toughness of the tested NiTi wires showed a significant increase following rapid-cycle autoclaving compared to the universal-cycle autoclaving which were intimately close to the values of the as-received archwires. Regarding spring back of the tested NiTi wires, no significant difference was detected following both autoclave cycles.
3. Average surface roughness (Sa) and Root mean square height (Sq) of the tested NiTi wires showed a significant decrease following rapid-cycle autoclaving compared to the universal-cycle autoclaving which were similar to the values of the as-received archwires.
4. On the clinical level, the mechanical properties and surface roughness of the tested NiTi wires were not notably altered by clinical use and autoclaving.

## Data Availability

I do not have any research data outside the submitted manuscript file. All data generated or analysed during this study are included in this published article.

## Abbreviations

NiTi: Nickel-titanium
TTR: Temperature transition range
AFM: Atomic force microscopy
Sa: Average surface roughness
